# The Effect of Acute Dehydration upon Muscle Strength Indices at Elite Karate Athletes: A Randomized Crossover Study

**DOI:** 10.3390/nu17091452

**Published:** 2025-04-25

**Authors:** Giannis Arnaoutis, Petros Neophytou

**Affiliations:** Laboratory of Nutrition & Clinical Dietetics, Department of Nutrition and Dietetics, School of Health Science and Education, Harokopio University of Athens, El. Venizelou Ave. 70, Kallithea, GR 17676 Athens, Greece; dp4422230@hua.gr

**Keywords:** dehydration, power, karate, combat sports, vertical jump, athletes, performance

## Abstract

**Background/Objectives**: Acute dehydration, commonly induced through fluid restriction and/or excessive sweating, is a common weight-cutting strategy among combat sport athletes. However, its impact on muscle strength and power remains a concern. The aim of the study was to evaluate the impact of 2% body mass reduction via dehydration on lower-limb strength and power in elite karate athletes. **Methods**: Fourteen male elite karate athletes completed two conditions: euhydrated (EUH) and dehydrated (DEH) (−2% body mass via 24-h fluid restriction). Performance was assessed using squat jump (SJ) and countermovement jump (CMJ) tests, along with isokinetic knee flexion and extension at 60, 180, and 300°/s. **Results**: Dehydration significantly reduced squat jump height (37.19 ± 3.69 vs. 39.34 ± 5.08 cm (EUH), *p* = 0.04), power output (2188.2 ± 307.2 vs. 2351.1 ± 347.2 W (EUH), *p* = 0.001), and knee extension and flexion strength at 60°/s (*p* = 0.018). CMJ height and higher-velocity knee flexion/extension were unaffected (*p* > 0.05). **Conclusions**: Acute dehydration impairs lower-body maximal force production at low velocities but has no significant effect on high velocity movements. Athletes and coaches should carefully manage hydration strategies when “cutting weight” to avoid any negative performance effects.

## 1. Introduction

Karate kumite, like many combat sports, is significantly influenced by the use of rapid weight loss (RWL) practices, which often involve acute dehydration in order for the athletes to compete in a lower weight categories [1]. RWL practices typically lead to a body mass reduction by 2–10% within the days prior to weigh-in [2]. Common methods include exercise-induced dehydration, fluid restriction, sauna, and low-calorie diets [1]. Despite the time frame between weigh-in and competition, many athletes fail to achieve optimal hydration status before competition [3].

The data indicate that there are different physiological profiles between grabbling and striking athletes [4]. The energy demands of karate kumite matches predominantly rely on anaerobic metabolism. Specifically, the anaerobic alactic pathway plays a crucial role for the execution of repeated rapid attacks and defenses, including powerful punches and explosive lower-limb kicks involving the shortening cycle (SSC), while grappling sports involve more upper body isometric strength to perform powerful lasting holds to control and unbalance the opponent [5]. Indicatively, Franchini et al. found that karate kumite relies heavily on anaerobic pathways, unlike grappling sports like judo, reporting three times the glycolytic and more than twice the ATP-PCr contribution [6]. Therefore, maximal velocity and explosive strength are two of the most important muscular factors for karate athletes [7,8] compared to high force/maximal strength demands of grappling sports. Moreover, longer-term anaerobic efforts seem to define successful grappling-based athletes, while superior athletes in striking sports like karate tend to show dominance in shorter-term measures [4]. Thus, there is a need for targeted studies in under-studied populations such as karate athletes, particularly to assess factors that may negatively impact key performance attributes and consequently affect karate kumite performance.

Although it is well documented that negative fluid balance impairs aerobic performance [9], its effect on anaerobic performance is less consistent, varying on the specific action measured [10,11]. A meta-analysis reported that a 3% reduction in body mass through dehydration negatively impacts strength and anaerobic power, but may enhance vertical jump performance, possibly due to reduced weight [12]. The aforementioned findings suggest that dehydration influences neuromuscular function through mechanisms that still remain unclear [13]. Moreover, dehydration under extreme conditions has been associated with serious health risks, including impaired endothelial function [14], acute kidney injury [15], electrolyte imbalances [16], and in some cases even death [2,17,18,19,20].

RWL practices are prevalent across various combat sports, including grappling (e.g., wrestling, judo, ju-jitsu), sticking (e.g., karate, taekwondo, boxing, kickboxing), and mixed martial arts (MMA) [1,21,22,23]. Alarmingly, athletes of all ages, including teenagers, and both genders engage repeatedly in these practices [1,24,25]. Despite concerns about potential health and performance decrements, a retrospective study analysing over 2100 MMA and boxing fights indicated that rapid weight gain after RWL correlates with success in combat competitions [26]. Moreover, research has shown that dehydration of ~3% body mass significantly reduced knee strength and endurance in competitive boxers [27], reduced cognitive function and taekwondo specific performance [28], decreased bench press mean propulsive velocity and counter movement jump, while having no effect on grip strength on boxers, wrestlers, and taekwondo athletes [3]. Furthermore, a 5% body mass reduction through dehydration has been shown to impair judo performance and grip strength [29]. However, Artioli et al. found no effect on judo performance or arm power when athletes had 4 h to rehydrate, highlighting the inconsistent relationship between hydration status and sport-specific performance [30]. These findings suggest that the effects of hydration balance on performance indicators in combat sports remain uncertain.

Preventive strategies are essential in sports to minimize injury risk and optimize long-term athletic performance. Early engagement in high-performance sports may lead to sport-specific physiological and muscular adaptations, underscoring the importance of tailored prevention approaches [31]. Athletes who begin high-performance training early often develop sport-specific neuromuscular adaptations that may affect performance and increase injury risk, even in the absence of trauma [32,33]. These adaptations can interact with factors like hydration status, making it important to examine how acute dehydration influences muscle function in elite athletes, particularly in sports where RWL practices are common. The current study adds to this perspective by introducing an additional preventive measure that may prove valuable in supporting athlete well-being and performance sustainability.

Despite the growing popularity of combat sports and the data on RWL prevalence [25,34,35,36,37], there is limited research on the effects of hypohydration and its relation to combat sports performance, especially on striking combat sports [3,38]. Moreover, there is a lack of studies involving elite athletes in-season. To our knowledge, this is the first study to examine the consequences of dehydration on strength and power in elite karate athletes. This study aims to quantify the impact of 2% body mass dehydration on lower limb strength and vertical jump performance in elite kumite athletes. We hypothesized that dehydration of 2% body mass would impair both strength and power.

## 2. Materials and Methods

### 2.1. Subjects

Fourteen elite kumite athletes, members of the Greek national karate team, participated in this randomized crossover study. Eligibility criteria included healthy, active male karatekas with a medal from a national championship and placement in a World Karate Federation competition within the last two years. Participants were required to be over 18 years old and weigh in the category of 67 to 84 kg. Exclusion criteria included the following: (i) tobacco or smoking product use; (ii) intake of any kind of supplement; (iii) adoption of extreme dietary or weight-loss methods (e.g., sauna use or excessive exercise under hot conditions); (iv) any known musculoskeletal injuries or chronic diseases. Their physical characteristics are presented in Table 1. The study was carried out in accordance with the principles of the Declaration of Helsinki. The experimental protocol was approved by the Ethics Committee of Harokopio University (Reg. No. 4317/23-10-2023).

### 2.2. Anthropometric Measurements

Body mass was assessed using a calibrated electronic scale (Seca GmbH, Hamburg, Germany) with participants wearing minimal clothing and no shoes. Height was measured using a stadiometer (Seca GmbH, Hamburg, Germany) to the nearest 0.1 cm, with the athletes’ weight equally distributed on their feet and their head, back, and buttocks on the vertical line of the height gauge. Body mass index (BMI) was consequently calculated as body weight in kilograms divided by the square of height in meters (kg/m^2^) based on the aforementioned measurements.

### 2.3. Body Composition Assessment

Body composition parameters, including fat-free mass, fat mass, body fat percentage, and bone mineral density, were evaluated using dual-energy X-ray absorptiometry (DXA) (DPX-MD, Lunar Corp., Madison, WI, USA). All DXA scans were performed in accordance with the manufacturer’s guidelines and were conducted by a trained technician to ensure precision and reliability. Participants were positioned supine during the scan, and measurements were taken under controlled conditions, including consistent temperature and low ambient light, to minimize variability. Quality control procedures were performed prior to each scanning session to verify device calibration and ensure the accuracy of measurements.

The day before each trial, participants were instructed to refrain from any strenuous exercise, consumption of alcohol or any caffeine-containing drinks, and standardize, as much as possible, their sleep. The subjects were also instructed to record their diet 24 h before their first visit. Compliance was verified by direct face to face follow-up and their diet record was copied and returned to the participants with instructions to follow the same diet before each subsequent visit. All testing and measurements were conducted in the morning after an overnight fast, separated by at least one week. For the performance tests, the subjects underwent one of the following interventions in a random order.

### 2.4. Hydration Status Protocol

Dehydration (DEH) was defined as a 2% reduction in body mass, the suggested threshold at which the negative effects of dehydration on athletic performance are commonly observed. The aforementioned level was achieved exclusively through 24 h of fluid restriction prior to testing. In more detail, upon arrival at the laboratory, participants emptied their bladders, and urine samples were collected for subsequent analysis. Baseline body weight was then recorded with the subjects wearing only their shorts. Body weight was compared to baseline values, and hydration status was assessed using urine specific gravity (USG) and urine color analysis. USG was measured in duplicate using a handheld clinical refractometer (Atago SUR-NE, Tokyo, Japan), with values ≥1.020 indicative of a hypohydrated state and values ≤1.019 indicative of a euhydrated state [39]. Urine color was further assessed by an experienced researcher who compared the color of the urine sample placed in a clear, glass 15-mL tube against white background, under fluorescent lighting, next to an original urine color scale, with values ≥4 indicating dehydration [40]. On the contrary, euhydrated (EUH) condition was ensured via water ingestion of at least 30 mL kg^−1^ of individual body mass during the previous day [41], along with an supplementary intake of 500 mL of water on the morning of the experiment. A computer-generated random number was used to randomly assign each participant to either EUH or DEH. Participants then performed the following performance tests after achieving the desired hydration status.

### 2.5. Vertical Jump Assessment

Upon confirmation of the desired hydration status, participants completed a standardized warm-up consisting of five minutes of low intensity (60–70 rpm) cycling on a stationary ergometer (Monark 839E; Monark Exercise, Vansbro, Sweden). Following the 5-min warm-up, and after another 5 min rest, the participants performed six vertical jumps: three counter-movement jumps (CMJs) and three squat jumps (SJs). Vertical jump performance was assessed using the MyJumpLab^®^ (MyJump 3) v4.2.7 application, a validated tool for measuring vertical jump height [42]. Video recordings were captured at 240 frames per second using an iPhone 13 Pro Max (Apple Inc., Cupertino, CA, USA), with the camera fixed 2 m perpendicular to the sagittal plane to ensure consistent analysis. Each jump was followed by a one-minute rest period, during which video analysis was conducted. Between the CMJs and SJs, a 5-min resting period was also provided. To minimize the risk of observational errors, two independent examiners reviewed the video recordings, and the examiner held the mobile at a standardized distance and height (0.7 m). The ICC (2.1) for jump height evaluation between the two raters yield a reliability value of 0.98. The mean jump height and power of the three trials was calculated for each jump type (CMJ and SJ) and used for subsequent analysis.

### 2.6. Isokinetic Testing

Subsequently and after a 10-min rest period, athletes were individually adjusted on the isokinetic dynamometer (BIODEX System 3 Pro, New York, NY, USA) seated upright with their hands by their sides. The trunk, waist, and experimental thigh were securely stabilized using specialized straps to minimize extraneous movements and ensure precise control of subsidiary motion during testing. The rotational axis of the lever arm was aligned with the anatomical center of rotation of the knee joint, facilitating accurate replication of natural joint mechanics. Subsequently, participants were instructed to perform five repetitions of knee extension and flexion through a 90° range of motion at three predetermined angular velocities (60°, 180°, and 300° per second), with intraclass correlation coefficients (ICC) reported at 0.99 [43] for reliability. All repetitions were conducted on the dominant leg, with the researchers providing explicit instructions to execute each repetition with maximum possible power. The measured parameters included peak torque, peak torque to bodyweight ratio, average power, acceleration time, and the agonist/antagonist ratio.

### 2.7. Statistical Analysis

All variables are presented as mean ± standard deviation because they were normally distributed. A Student’s paired *t*-test analysis was employed to evaluate the effect of hydration status (EUH and DEH) in the main study variables (muscle power and muscle strength). To estimate the sample size, G*Power v. 3.1.9.7 was used. Based on the observed effect size for squat jump (SJ) height (Cohen’s d = 0.6), and a probability level of 0.05, our sample of 14 athletes yields a statistical power of 0.82, which is considered adequate for detecting meaningful differences in performance metrics like jump height and muscle power. Significance was accepted at the <0.05 level. All statistical analyses were performed using SPSS 21 (SPSS Inc., Chicago, IL, USA).

## 3. Results

### 3.1. Hydration Status

All participants adhered to each hydration protocol, with no deviations or dietary changes during the study period. Urine Specific Gravity (USG) values were recorded to verify hydration status in both the EUH and DEH conditions. In EUH condition, USG values ranged from 1.005 to 1.017, confirming adequate hydration. In contrast, the 24-h water restriction led to a body mass reduction of −1.9 ± 0.1%, with USG values ranging from 1.028 to 1.035, confirming successful dehydration. These values align with the established thresholds for euhydration and hypohydration (USG ≥ 1.020) [40].

### 3.2. Vertical Jump

**Figure 1:**
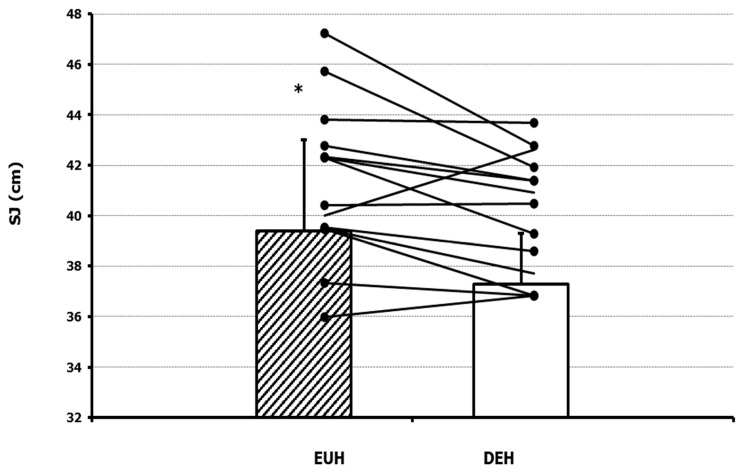
Individual Squat Jump (SJ) height data in euhydrated (EUH) and dehydrated (DEH) condition. Bars and standard error represent the mean and standard error of the mean on each condition, while lines represent individual subjects. * *p*-value < 0.05.

Vertical jump height and power outcomes for both countermovement jumps (CMJ) and squat jumps (SJ) are presented in Table 2. SJ height demonstrated a significant difference between the EUH and DEH conditions, showing a 5.4% reduction (39.34 ± 5.08 vs. 37.19 ± 3.69 cm; *p* = 0.04) (Figure 1). In contrast, CMJ height demonstrated a non-significant reduction between the two conditions. Specifically, mean jump heights were 42.93 ± 4.92 cm in the EUH condition and 41.85 ± 5.06 cm in the DEH condition (*p* = 0.256).

For jump power, SJ power was significantly impaired in the DEH condition, showing a 7.1% reduction compared to EUH (2188.2 ± 307.2 vs. 2351.1 ± 347.2 W; *p* = 0.001). Conversely, CMJ values were 2536 ± 396 W in the EUH condition and 2448 ± 422 W in the DEH condition, but this decrease did not reach statistical significance (*p* = 0.169).

### 3.3. Isokinetic Dynamometry

Knee extension and flexion strength were evaluated at angular velocities of 60°/s, 180°/s, and 300°/s (Table 3). A statistically significant reduction in mean power of knee extension and flexion was observed at 60°/s with values of 158.02 ± 28.57 W for EUH vs. 164.91 ± 26.69 W for DEH; and 99.10 ± 16.31 vs. 96.65 ± 18.87 W, respectively, *p* = 0.018. However, no statistically significant differences were found in knee extension or flexion strength at the higher angular velocities of 180°/s and 300°/s between hydration states or any other measured parameter nor at acceleration time (all *p* > 0.05).

## 4. Discussion

The primary objective of this study was to examine the effects of acute dehydration (−2% body mass) on lower-body strength and power in elite karate athletes. Our findings provide novel insights into the effects of acute dehydration on neuromuscular performance in a population that has been understudied in scientific research. While the impact of dehydration on endurance performance has been extensively studied [9,44], there is a notable lack of data on its influence upon explosive power and strength, particularly in in striking combat sports like karate [3,38]. Our results revealed a significant decline in squat jump (SJ) performance and knee extensor/flexion strength at low angular velocities (60°/s) under dehydrated conditions. However, no significant impairment was observed in countermovement jump (CMJ) performance or isokinetic strength at higher angular velocities (180°/s and 300°/s). This suggests that dehydration primarily affects maximal force production at slower contraction speeds while sparing high-velocity movements crucial for kumite performance.

The decrease in SJ performance under dehydrated conditions aligns with previous research indicating that maximal concentric force production is sensitive to fluid loss [45]. It should, however, be mentioned that 2 out of the 14 athletes improved SJ, an observation that may reflect individual adaptations or familiarity with RWL. Since the SJ lacks a preloading phase, it relies entirely on concentric muscle actions, which appear more susceptible to dehydration-related impairments. Contrarywise, the CMJ involves a stretch-shortening cycle (SSC), allowing for greater utilization of stored elastic energy and neuromuscular efficiency, which may mitigate dehydration-related deficits, while the greater uptake of muscle slack and the buildup of stimulation during the countermovement in a CMJ may also partially explain the differences between countermovement and squat jump performances [46,47]. However, neural adaptability (e.g., altered motor unit recruitment) and/or technical differences in jump execution cannot be ruled out. Our findings suggest that karate-specific explosive actions, which repeatedly incorporate stretch-shortening mechanisms, may remain largely unaffected under mild dehydration conditions.

Similarly, the reduction in knee extensor/flexor strength at 60°/s suggests that dehydration impairs force production during slow, continuous movements, where sustained force output is required [45,48]. This finding is consistent with studies indicating that reduction of intracellular muscle water affects the muscles contractile capacity, limiting maximal force generation in slower, sustained movements [11,49,50]. These results are in line with a recent systematic review that concluded that acute dehydration significantly reduces maximal strength during slow-speed isokinetic contractions [48]. Though the reasons for this selective impairment remain unclear, they may involve: (i) the disruption of electrolyte balance, which in turn impairs nerve conduction and muscle fiber recruitment—particularly for slow-twitch fibers used in sustained activity; and (ii) impaired motor unit recruitment, as evidenced by decreased electromyographic (EMG) activity during maximal contractions, which is critical for maintaining muscle performance in activities requiring prolonged effort [51,52]. Nevertheless, the lack of significant impairment at 180°/s and 300°/s indicates that rapid, high-velocity muscle contractions are less dependent on hydration status. This differential impact on low- vs. high-speed movements may be attributed to dehydration-induced reductions in intracellular water content, resulting in cellular shrinkage and impaired cross-bridge cycling. Additionally, reactive oxygen species (ROS)-mediated inhibition of the SERCA pump may disrupt calcium re-uptake, prolonging muscle relaxation times [52]. Together, these mechanisms are likely to contribute to the aforementioned declines in low-velocity strength, particularly during sustained or repeated contractions. Since karate kumite performance is primarily defined by quick bursts of power rather than prolonged force production, isokinetic strength at higher velocities may be a more relevant performance metric compared to strength at slow speeds [53,54].

Kumite has been described as a sport whose predominant energy source is aerobic metabolism [5,7]. Previous research demonstrated a decrease in aerobic performance under hypohydrated conditions [9,55,56]. The findings of this study suggest that the repeated execution of maximal effort punches and kicks may be unaffected if the loss of water is moderate (<3%). It has been established that jump height is directly correlated with punching power [57,58,59], so it is safe to assume that the power advantage when competing in a lower weight category does not diminish after incomplete rehydration post RWL as shown in previous research conducted on combat sports [3,10,30,60]. This is particularly relevant for combat sport practitioners who undergo RWL and do not fully recover by competition time or in between consecutive matches. Effective post weight-in rehydration strategies are critical for optimizing competition performance through restoration of plasma volume, electrolyte balance, and muscle function, all of which are essential for peak athletic performance [61]. Failure to adequately rehydrate can lead to decreased strength, endurance, and cognitive function, negatively impacting competitive outcomes. Therefore, structured rehydration protocols—including fluid intake with electrolytes and carbohydrates—are vital for ensuring physiological recovery and readiness for competition [62].

It is also important to recognize that a very high degree of variation exists in performance of a sport skill or task and that any alteration in performance could vary depending on the type of skill or task being performed [11,13]. As mentioned previously, most research on combat sports performance and hydration focused heavily on grappling related sports in which demands differ significantly from striking sports such as karate [6]. Furthermore, the dehydration protocol, the magnitude of dehydration, and the testing methods vary from study to study [3,27,29,30,36,63]. Based on the current available literature on combat sports and dehydration, the results seem inconsistent, with studies finding significant reduction in performance [27,29,64], while others show no effect [65,66,67]. The limited experimental research on strikers to date indicates only a decline in performance [3,27]. While familiarization with dehydration has been shown to mitigate performance decreases in endurance [68], its effect on strength remains uncertain. Combat sport athletes frequently undergo dehydration [36], yet the results of this study clearly demonstrate a reduction in strength.

From a practical standpoint, maintenance of a euhydrated condition is important for overall health and recovery, as excessive dehydration (>3% body mass) can negatively impact endurance, perceived exertion, thermoregulation, and cognitive function [69,70,71]. It should also be highlighted that repetitive RWL methods and/or extreme weight gain efforts can lead to serious health risks, ranging from compromised immune function, increased muscle damage markers, risk of developing eating disorders [62], and reaching to acute kidney injury [72] and even kidney dysfunction [17]. However, the present results suggest that mild hypohydration (−2% body mass) may not significantly compromise kumite performance despite a reduction in maximal strength, as it does not impair the high-speed, explosive actions that define the sport. This classifies mild dehydration as a potentially effective strategy for competing in a lower weight category without severely diminishing performance. Combat sports that rely more on sustained maximal effort should consider these findings when implementing fluid restriction as a weight-cutting method.

Success in combat sports performance is multifactorial [6]. The complex nature of these sports makes it challenging to identify the exact mechanisms responsible for performance reductions observed in various studies. By addressing this research gap, the present study enhances our understanding of how RWL strategies, commonly used by karate and other combat sport athletes, may compromise their ability to generate explosive power. The findings of the present study suggest that while mild dehydration via fluid restriction reduces maximal force production, the key explosive actions essential for kumite performance remain largely unaffected. This insight is crucial for athletes and coaches seeking to balance the demands of weight category restrictions while maintaining peak physical performance.

Despite the strengths of this study—such as its focus on a rarely studied athletic population (elite in-season karate athletes) and its examination of dehydration’s impact on explosive power and strength, performance characteristics which are often overlooked, certain limitations should be acknowledged. The homogeneity (elite male karate athletes) and the relatively limited size of the sample (noting the extremely rare, understudied, and difficult-to-recruit population) limits the generalizability of the findings, particularly to female athletes, who also engage in rapid weight loss practices [24,25]. The absence of female participants precludes consideration of potential sex-specific responses to dehydration, including the influence of hormonal fluctuations. Progesterone-mediated fluid retention during the luteal phase of the menstrual cycle may impact hydration status and performance outcomes, underscoring the need for future research in female combat athletes [73]. Additionally, studies should focus on lower-level competitors to investigate how athletic levels may influence performance responses. Finally, while the use of the MyJumpLab application for assessing vertical jumps is validated [42,74], it involves manual video analysis, which may introduce minor observational errors. To mitigate this, two independent examiners reviewed the data to ensure accuracy.

## 5. Conclusions

In conclusion, this study demonstrates that acute dehydration (−2% body mass) impairs squat jump height and low-speed isokinetic strength, while countermovement jump (CMJ and high-speed velocity force production–crucial for kumite performance–remained unaffected in elite karate athletes. Nonetheless, our findings contribute to the growing body of evidence suggesting that mild dehydration may selectively impact certain strength parameters while leaving others unaffected, particularly in sports that rely on explosive power. While maintaining proper hydration is essential for overall health and recovery, carefully planned fluid restriction as a weight-cutting strategy may be effective without significantly compromising sport-specific performance. However, the underlying mechanisms behind this selective effect remain unclear. Further research is needed to develop hydration strategies tailored to the unique demands of striking combat sports and to explore the long-term implications of repeated dehydration practices in elite athletes.

## Figures and Tables

**Table 1:** Participant Characteristics.

Characteristics	*n* = 14
Age (years)	23.4 ± 3.5
Years of training	11.5 ± 3.5
Height (cm)	175 ± 6.5
Body Mass (kg)	76.6 ± 6.5
BMI (kg/m^2^)	25.2 ± 2.1
Body Fat (%)	13.1 ± 4.3
Fat mass (kg)	9.4 ± 3.6
Fat-Free Mass (kg)	62.6 ± 5.2
BMD (g/cm²)	1.348 ± 0.07

Values are means ± standard deviation. BMI, body mass index. BMD, bone mineral density.

**Table 2:** Vertical Jump Measurements.

	EUH	DEH	*p*-Value
CMJ Height (cm)	42.93 ± 4.92	41.85 ± 5.06	0.256
CMJ Power (W)	2536 ± 396	2448 ± 422	0.169
SJ Height (cm)	39.34 ± 5.08 *	37.19 ± 3.69	0.04
SJ Power (W)	2351.1 ± 347.2 †	2188.2 ± 307.2	0.001

Values are mean ± standard deviation. * = significantly different from dehydrated condition, at *p* < 0.05 level. † = significantly different from dehydrated condition, at *p* < 0.01 level.

**Table 3:** Isokinetic Dynamometer Results.

		Peak Torque (N-M)	Peak TQ/BW (%)	Avg Power (W)	Acceleration Time (ms)
Extension 60°	Euhydrated	227.8 ± 35.6	290.3 ± 54.2	164.91 ± 26.69 *	22.14 ± 15.78
	Dehydrated	231.7 ± 37.8	306.2 ± 30.1	158.02 ± 28.57	28.57 ± 10.99
Extension 180°	Euhydrated	163.51 ± 22.15	210.5 ± 42.9	309.4 ± 40.1	46.43 ± 12.16
	Dehydrated	162.91 ± 18.68	216.1 ± 12.6	297.6 ± 41.8	46.43 ± 10.08
Extension 300°	Euhydrated	133.01 ± 17.32	189.6 ± 58.3	342.9 ± 87.8	64.45 ± 10.28
	Dehydrated	130.04 ± 14.93	173.7 ± 14.5	346.3 ± 36.6	60.00 ± 7.84
Flexion 60°	Euhydrated	128.1 ± 21.9	164.11 ± 34.99	99.10 ± 16.31 †	42.14 ± 8.93
	Dehydrated	127.1 ± 38.6	178.40 ± 23.38	96.65 ± 18.87	45.71 ± 11.58
Flexion 180°	Euhydrated	96.64 ± 12.74	124.14 ± 24.22	182.39 ± 27.42	73.50 ± 11.47
	Dehydrated	95.06 ± 27.43	136.06 ± 17.75	181.99 ± 36.47	79.29 ± 15.42
Flexion 300°	Euhydrated	83.75 ± 9.28	104.01 ± 27.38	212.97 ± 28.43	98.57 ± 10.27
	Dehydrated	87.08 ± 12.58	116.57 ± 14.02	207.68 ± 36.68	100.00 ± 16.17

Values are mean ± standard deviation. * = significantly different from dehydrated condition at *p* < 0.05 level. † = significantly different from dehydrated condition, at *p* < 0.05 level.

## Data Availability

The data presented in this study are available for non-commercial scientific inquiry and educational use upon request from the corresponding author.

## References

[1] Brito C.J., Roas A.F.C.M., Brito I.S.S., Marins J.C.B., Córdova C., Franchini E. (2012). Methods of Body Mass Reduction by Combat Sport Athletes. Int. J. Sport. Nutr. Exerc. Metab..

[2] Artioli G.G., Saunders B., Iglesias R.T., Franchini E. (2016). It Is Time to Ban Rapid Weight Loss from Combat Sports. Sports Med..

[3] Pallarés J.G., Martínez-Abellán A., López-Gullón J.M., Morán-Navarro R., De la Cruz-Sánchez E., Mora-Rodríguez R. (2016). Muscle Contraction Velocity, Strength and Power Output Changes Following Different Degrees of Hypohydration in Competitive Olympic Combat Sports. J. Int. Soc. Sports Nutr..

[4] James L.P., Haff G.G., Kelly V.G., Beckman E.M. (2016). Towards a Determination of the Physiological Characteristics Distinguishing Successful Mixed Martial Arts Athletes: A Systematic Review of Combat Sport Literature. Sports Med..

[5] Beneke R., Beyer T., Jachner C., Erasmus J., Hütler M. (2004). Energetics of Karate Kumite. Eur. J. Appl. Physiol..

[6] Franchini E. (2023). Energy System Contributions during Olympic Combat Sports: A Narrative Review. Metabolites.

[7] Doria C., Veicsteinas A., Limonta E., Maggioni M.A., Aschieri P., Eusebi F., Fanò G., Pietrangelo T. (2009). Energetics of Karate (Kata and Kumite Techniques) in Top-Level Athletes. Eur. J. Appl. Physiol..

[8] Katić R., Blazević S., Zagorac N. (2010). The Impact of Basic Motor Abilities on the Specific Motoricity Performance in Elite Karateka. Coll. Antropol..

[9] Deshayes T.A., Jeker D., Goulet E.D.B. (2020). Impact of Pre-Exercise Hypohydration on Aerobic Exercise Performance, Peak Oxygen Consumption and Oxygen Consumption at Lactate Threshold: A Systematic Review with Meta-Analysis. Sports Med..

[10] Barley O.R., Iredale F., Hopper A., Abbiss C.R. (2018). Repeat Effort Performance Is Reduced 24 Hours After Acute Dehydration in Mixed Martial Arts Athletes. J. Strength Cond. Res..

[11] Cheuvront S.N., Kenefick R.W. (2014). Dehydration: Physiology, Assessment, and Performance Effects. Compr. Physiol..

[12] Savoie F.-A., Kenefick R.W., Ely B.R., Cheuvront S.N., Goulet E.D.B. (2015). Effect of Hypohydration on Muscle Endurance, Strength, Anaerobic Power and Capacity and Vertical Jumping Ability: A Meta-Analysis. Sports Med..

[13] Brechney G.C., Cannon J., Goodman S.P. (2022). Effects of Weight Cutting on Exercise Performance in Combat Athletes: A Meta-Analysis. Int. J. Sports Physiol. Perform..

[14] Arnaoutis G., Kavouras S.A., Stratakis N., Likka M., Mitrakou A., Papamichael C., Sidossis L.S., Stamatelopoulos K. (2017). The Effect of Hypohydration on Endothelial Function in Young Healthy Adults. Eur. J. Nutr..

[15] Chapman C.L., Holt S.M., O’Connell C.T., Brazelton S.C., Howells W.A.B., Medved H.N., Reed E.L., Needham K.W., Halliwill J.R., Minson C.T. (2023). Acute Kidney Injury Biomarkers and Hydration Assessments Following Prolonged Mild Hypohydration in Healthy Young Adults. Am. J. Physiol. Renal Physiol..

[16] Burke L.M., Slater G.J., Matthews J.J., Langan-Evans C., Horswill C.A. (2021). ACSM Expert Consensus Statement on Weight Loss in Weight-Category Sports. Curr. Sports Med. Rep..

[17] Kasper A.M., Crighton B., Langan-Evans C., Riley P., Sharma A., Close G.L., Morton J.P. (2019). Case Study: Extreme Weight Making Causes Relative Energy Deficiency, Dehydration, and Acute Kidney Injury in a Male Mixed Martial Arts Athlete. Int. J. Sport. Nutr. Exerc. Metab..

[18] Murugappan K.R., Cocchi M.N., Bose S., Neves S.E., Cook C.H., Sarge T., Shaefi S., Leibowitz A. (2019). Case Study: Fatal Exertional Rhabdomyolysis Possibly Related to Drastic Weight Cutting. Int. J. Sport. Nutr. Exerc. Metab..

[19] Hoey J. (1998). Wrestling Hyperthermia and Dehydration. CMAJ.

[20] (1998). Centers for Disease Control and Prevention (CDC) Hyperthermia and Dehydration-Related Deaths Associated with Intentional Rapid Weight Loss in Three Collegiate Wrestlers—North Carolina, Wisconsin, and Michigan, November–December 1997. MMWR Morb. Mortal. Wkly. Rep..

[21] Matthews J.J., Stanhope E.N., Godwin M.S., Holmes M.E.J., Artioli G.G. (2019). The Magnitude of Rapid Weight Loss and Rapid Weight Gain in Combat Sport Athletes Preparing for Competition: A Systematic Review. Int. J. Sport. Nutr. Exerc. Metab..

[22] Reale R., Slater G., Cox G.R., Dunican I.C., Burke L.M. (2018). The Effect of Water Loading on Acute Weight Loss Following Fluid Restriction in Combat Sports Athletes. Int. J. Sport. Nutr. Exerc. Metab..

[23] Barley O.R., Chapman D.W., Abbiss C.R. (2018). Weight Loss Strategies in Combat Sports and Concerning Habits in Mixed Martial Arts. Int. J. Sports Physiol. Perform..

[24] Ceylan B., Balci S.S. (2023). Dehydration and Rapid Weight Gain Between Weigh-in and Competition in Judo Athletes: The Differences between Women and Men. Res. Sports Med..

[25] Evans C., Stull C., Sanders G., Ricci A., French D., Antonio J., Peacock C.A. (2023). Weight Cutting in Female UFC Fighters. J. Int. Soc. Sports Nutr..

[26] Baribeau V., Kirk C., Le D.Q., Bose A., Mueller A., French D., Sarge T., Langan-Evans C., Reale R., Murugappan K.R. (2023). Rapid Weight Gain and Weight Differential Predict Competitive Success in 2100 Professional Combat-Sport Athletes. Int. J. Sports Physiol. Perform..

[27] Zubac D., Šimunič B., Buoite Stella A., Morrison S.A. (2020). Neuromuscular Performance after Rapid Weight Loss in Olympic-Style Boxers. Eur. J. Sport. Sci..

[28] Zheng A.-C., He C.-S., Lu C.-C., Hung B.-L., Chou K.-M., Fang S.-H. (2024). The Cognitive Function and Taekwondo-Specific Kick Performance of Taekwondo Athletes at Different Hydration Statuses. Int. J. Sports Physiol. Perform..

[29] Ceylan B., Kons R.L., Detanico D., Šimenko J. (2022). Acute Dehydration Impairs Performance and Physiological Responses in Highly Trained Judo Athletes. Biology.

[30] Artioli G.G., Iglesias R.T., Franchini E., Gualano B., Kashiwagura D.B., Solis M.Y., Benatti F.B., Fuchs M., Lancha Junior A.H. (2010). Rapid Weight Loss Followed by Recovery Time Does Not Affect Judo-Related Performance. J. Sports Sci..

[31] Brenner J.S., COUNCIL ON SPORTS MEDICINE AND FITNESS (2016). Sports Specialization and Intensive Training in Young Athletes. Pediatrics.

[32] Xu D., Zhou H., Quan W., Ma X., Chon T.-E., Fernandez J., Gusztav F., Kovács A., Baker J.S., Gu Y. (2024). New Insights Optimize Landing Strategies to Reduce Lower Limb Injury Risk. Cyborg Bionic Syst..

[33] Chiaramonte R., Testa G., Russo A., Buccheri E., Milana M., Prezioso R., Pavone V., Vecchio M. (2024). Damage for Gain: The Useful Damage of the Pitcher’s Paradox. Heliyon.

[34] Barley O.R., Chapman D.W., Abbiss C.R. (2019). The Current State of Weight-Cutting in Combat Sports-Weight-Cutting in Combat Sports. Sports.

[35] Brechney G.C., Chia E., Moreland A.T. (2021). Weight-Cutting Implications for Competition Outcomes in Mixed Martial Arts Cage Fighting. J. Strength Cond. Res..

[36] Dugonjić B., Krstulović S., Kuvačić G. (2019). Rapid Weight Loss Practices in Elite Kickboxers. Int. J. Sport. Nutr. Exerc. Metab..

[37] Daniele G., Weinstein R.N., Wallace P.W., Palmieri V., Bianco M. (2016). Rapid Weight Gain in Professional Boxing and Correlation with Fight Decisions: Analysis from 71 Title Fights. Phys. Sportsmed..

[38] Martínez-Aranda L.M., Sanz-Matesanz M., Orozco-Durán G., González-Fernández F.T., Rodríguez-García L., Guadalupe-Grau A. (2023). Effects of Different Rapid Weight Loss Strategies and Percentages on Performance-Related Parameters in Combat Sports: An Updated Systematic Review. Int. J. Environ. Res. Public. Health.

[39] Armstrong L.E., Maresh C.M., Castellani J.W., Bergeron M.F., Kenefick R.W., LaGasse K.E., Riebe D. (1994). Urinary Indices of Hydration Status. Int. J. Sport. Nutr..

[40] Armstrong L.E., Soto J.A., Hacker F.T., Casa D.J., Kavouras S.A., Maresh C.M. (1998). Urinary Indices during Dehydration, Exercise, and Rehydration. Int. J. Sport. Nutr..

[41] Kavouras S.A., Armstrong L.E., Maresh C.M., Casa D.J., Herrera-Soto J.A., Scheett T.P., Stoppani J., Mack G.W., Kraemer W.J. (2006). Rehydration with Glycerol: Endocrine, Cardiovascular, and Thermoregulatory Responses during Exercise in the Heat. J. Appl. Physiol..

[42] Bishop C., Jarvis P., Turner A., Balsalobre-Fernandez C. (2022). Validity and Reliability of Strategy Metrics to Assess Countermovement Jump Performance Using the Newly Developed My Jump Lab Smartphone Application. J. Hum. Kinet..

[43] Drouin J.M., Valovich-mcLeod T.C., Shultz S.J., Gansneder B.M., Perrin D.H. (2004). Reliability and Validity of the Biodex System 3 pro Isokinetic Dynamometer Velocity, Torque and Position Measurements. Eur. J. Appl. Physiol..

[44] Goulet E.D.B. (2013). Effect of Exercise-Induced Dehydration on Endurance Performance: Evaluating the Impact of Exercise Protocols on Outcomes Using a Meta-Analytic Procedure. Br. J. Sports Med..

[45] Hayes L.D., Morse C.I. (2010). The Effects of Progressive Dehydration on Strength and Power: Is There a Dose Response?. Eur. J. Appl. Physiol..

[46] Van Hooren B., Zolotarjova J. (2017). The Difference Between Countermovement and Squat Jump Performances: A Review of Underlying Mechanisms With Practical Applications. J. Strength Cond. Res..

[47] Van Hooren B., Bosch F. (2016). Influence of Muscle Slack on High-Intensity Sport Performance: A Review. Strength Cond. J..

[48] Francisco R., Jesus F., Santos P., Trbovšek P., Moreira A.S., Nunes C.L., Alvim M., Sardinha L.B., Lukaski H., Mendonca G.V. (2024). Does Acute Dehydration Affect the Neuromuscular Function in Healthy Adults?-A Systematic Review. Appl. Physiol. Nutr. Metab..

[49] Lorenzo I., Serra-Prat M., Yébenes J.C. (2019). The Role of Water Homeostasis in Muscle Function and Frailty: A Review. Nutrients.

[50] Judelson D.A., Maresh C.M., Anderson J.M., Armstrong L.E., Casa D.J., Kraemer W.J., Volek J.S. (2007). Hydration and Muscular Performance: Does Fluid Balance Affect Strength, Power and High-Intensity Endurance?. Sports Med..

[51] Rodrigues R., Baroni B.M., Pompermayer M.G., de Oliveira Lupion R., Geremia J.M., Meyer F., Vaz M.A. (2014). Effects of Acute Dehydration on Neuromuscular Responses of Exercised and Nonexercised Muscles after Exercise in the Heat. J. Strength. Cond. Res..

[52] Uddin N., Tallent J., Patterson S.D., Goodall S., Waldron M. (2022). Corticospinal and Peripheral Responses to Heat-Induced Hypo-Hydration: Potential Physiological Mechanisms and Implications for Neuromuscular Function. Eur. J. Appl. Physiol..

[53] Güler M., Ramazanoglu N. (2018). Evaluation of Physiological Performance Parameters of Elite Karate-Kumite Athletes by the Simulated Karate Performance Test. Univers. J. Educ. Res..

[54] Sbriccoli P., Camomilla V., Di Mario A., Quinzi F., Figura F., Felici F. (2010). Neuromuscular Control Adaptations in Elite Athletes: The Case of Top Level Karateka. Eur. J. Appl. Physiol..

[55] Sawka M.N., Cheuvront S.N., Kenefick R.W. (2012). High Skin Temperature and Hypohydration Impair Aerobic Performance. Exp. Physiol..

[56] Goulet E.D.B. (2011). Effect of Exercise-Induced Dehydration on Time-Trial Exercise Performance: A Meta-Analysis. Br. J. Sports Med..

[57] Kons R.L., Ache-Dias J., Detanico D., Barth J., Dal Pupo J. (2018). Is Vertical Jump Height an Indicator of Athletes’ Power Output in Different Sport Modalities?. J. Strength. Cond. Res..

[58] Loturco I., Artioli G.G., Kobal R., Gil S., Franchini E. (2014). Predicting Punching Acceleration from Selected Strength and Power Variables in Elite Karate Athletes: A Multiple Regression Analysis. J. Strength. Cond. Res..

[59] Loturco I., Nakamura F.Y., Artioli G.G., Kobal R., Kitamura K., Cal Abad C.C., Cruz I.F., Romano F., Pereira L.A., Franchini E. (2016). Strength and Power Qualities Are Highly Associated With Punching Impact in Elite Amateur Boxers. J. Strength. Cond. Res..

[60] Serfass R.C., Stull G.A., Alexander J.F., Ewing J.L. (1984). The Effects of Rapid Weight Loss and Attempted Rehydration on Strength and Endurance of the Handgripping Muscles in College Wrestlers. Res. Q. Exerc. Sport.

[61] Reale R., Slater G., Burke L.M. (2017). Acute-Weight-Loss Strategies for Combat Sports and Applications to Olympic Success. Int. J. Sports Physiol. Perform..

[62] dos Santos D.F.C., Yang W.-H., Franchini E. (2024). A Scoping Review of Rapid Weight Loss in Judo Athletes: Prevalence, Magnitude, Effects on Performance, Risks, and Recommendations. Phys. Act. Nutr..

[63] Koral J., Dosseville F. (2009). Combination of Gradual and Rapid Weight Loss: Effects on Physical Performance and Psychological State of Elite Judo Athletes. J. Sports Sci..

[64] Hall C.J., Lane A.M. (2001). Effects of Rapid Weight Loss on Mood and Performance among Amateur Boxers. Br. J. Sports Med..

[65] Cheuvront S.N., Carter R., Haymes E.M., Sawka M.N. (2006). No Effect of Moderate Hypohydration or Hyperthermia on Anaerobic Exercise Performance. Med. Sci. Sports Exerc..

[66] Hosick P.A., Sheris A., Alencewicz J.S., Matthews E.L. (2020). Mild Dehydration Following Voluntary Water Intake Reduction Does Not Affect Anaerobic Power Performance. J. Sports Med. Phys. Fit..

[67] Mendes S.H., Tritto A.C., Guilherme J.P.L.F., Solis M.Y., Vieira D.E., Franchini E., Lancha A.H., Artioli G.G. (2013). Effect of Rapid Weight Loss on Performance in Combat Sport Male Athletes: Does Adaptation to Chronic Weight Cycling Play a Role?. Br. J. Sports Med..

[68] Fleming J., James L.J. (2014). Repeated Familiarisation with Hypohydration Attenuates the Performance Decrement Caused by Hypohydration during Treadmill Running. Appl. Physiol. Nutr. Metab..

[69] Liska D., Mah E., Brisbois T., Barrios P.L., Baker L.B., Spriet L.L. (2019). Narrative Review of Hydration and Selected Health Outcomes in the General Population. Nutrients.

[70] Wittbrodt M.T., Millard-Stafford M. (2018). Dehydration Impairs Cognitive Performance: A Meta-Analysis. Med. Sci. Sports Exerc..

[71] Deshayes T.A., Pancrate T., Goulet E.D.B. (2022). Impact of Dehydration on Perceived Exertion during Endurance Exercise: A Systematic Review with Meta-Analysis. J. Exerc. Sci. Fit..

[72] Lakicevic N., Paoli A., Roklicer R., Trivic T., Korovljev D., Ostojic S.M., Proia P., Bianco A., Drid P. (2021). Effects of Rapid Weight Loss on Kidney Function in Combat Sport Athletes. Medicina.

[73] McNulty K.L., Elliott-Sale K.J., Dolan E., Swinton P.A., Ansdell P., Goodall S., Thomas K., Hicks K.M. (2020). The Effects of Menstrual Cycle Phase on Exercise Performance in Eumenorrheic Women: A Systematic Review and Meta-Analysis. Sports Med..

[74] Gençoğlu C., Ulupınar S., Özbay S., Turan M., Savaş B.Ç., Asan S., İnce İ. (2023). Validity and Reliability of “My Jump App” to Assess Vertical Jump Performance: A Meta-Analytic Review. Sci. Rep..

